# Impact of Photoperiod Length and Treatment with Exogenous Melatonin during Pregnancy on Chemical Composition of Sheep’s Milk

**DOI:** 10.3390/ani10101721

**Published:** 2020-09-23

**Authors:** Edyta Molik, Michał Błasiak, Henryk Pustkowiak

**Affiliations:** 1 Department of Animal Nutrition and Biotechnology, and Fisheries, Faculty of Animal Sciences, University of Agriculture in Krakow, al. A. Mickiewicza 24/28, 30–059 Krakow, Poland; mb2-red@o2.pl; 2 Department of Genetics and Animal Breeding and Ethology, Faculty of Animal Sciences, University of Agriculture in Krakow, al. A. Mickiewicza 24/28, 30–059 Krakow, Poland; henryk.pustkowiak@urk.edu.pl

**Keywords:** milk, photoperiod, melatonin

## Abstract

**Simple Summary:**

The high content of valuable nutrients and biologically active substances in sheep’s milk positively affects the dietary value of this milk and products derived from it. Studies carried out so far indicate that for ewes, the date (season) of lambing, and thus also the period when lactation is initiated influences the biochemical parameters of milk. Results from the experiment carried out by the authors can serve as foundation for usage of sheep’s milk in so-called nutritional programming in the treatment of many human diseases. In seasonal animals such as sheep, length of day and melatonin signal have a significant impact on lactation parameters and chemical composition of milk. Sheep’s milk offers a number of health-promoting properties and can be used in prevention of many human diseases. An important factor contributing to these beneficial properties of sheep’s milk is its fat content, which is rich in bioactive substances, such as CLA (Conjugated Linoleic Acid), with antioxidant properties.

**Abstract:**

The aim of the study was to determine the effect of photoperiod and exogenous melatonin on milk yield and chemical composition of sheep’s milk. Sheep (*n* = 60) were randomly divided into three groups: lambing in February (Group 1—*n* = 20), lambing in June (Group 2—*n* = 20), and lambing in June and treated with subcutaneous melatonin implants (Group 3—*n* = 20). Milk yield was higher for Group 1 and Group 2 than for Group 3 (*p* < 0.01). The milk of ewes of Groups 2 and 3 had a significantly (*p* < 0.01) higher content of dry matter, protein, and fat. Group 3 sheep’s milk contained significantly more (*p* < 0.01) of SFA (Saturated Fatty Acids). The highest content of MUFA (Monounsaturated Fatty Acids) and PUFA (Polyunsaturated Fatty Acids) was found in the samples collected from Group 1, the lowest was in the milk of Group 3 animals. The highest (*p* < 0.01) CLA, content was identified in the milk of Group 1, while the lowest was recorded for the milk obtained from sheep treated with exogenous melatonin (Group 3). The experiment carried out has shown that day length and treatment with exogenous melatonin modulate the chemical composition of milk.

## 1. Introduction

Sheep, as animals with marked breeding seasonality, react to changes in length of day, which serve as the main signal for onset of the period of sexual activity. In these animals, photoperiod length has a significant impact on secretion of melatonin and prolactin [1,2,3]. In short-day animals exposed to decreasing photoperiod length, melatonin levels were found to increase while prolactin secretion was in decline [2,3]. Changes in prolactin secretion in lactating sheep influence the amount of milk produced [4,5,6]. Sheep’s milk is richest in vaccenic acid, precursor of CLA, and this content is also subject to seasonal fluctuations from—0.54% in winter to 1.28% in summer [7,8]. Milk of sheep contains important amino-acids (such as lysine, histidine, serine, and valine) and peptides showing biological activity (casokinines, lactoferroxins, lactorphins) [9]. This milk is characterized by a higher content of minerals and macronutrients (calcium, potassium, phosphorus, chlorine, sodium) than the milk from other species [10]. Due to its chemical parameters, sheep’s milk is a product that already in its original raw form has particularly beneficial effects on the health of consumers. Health-promoting properties of this milk and its products are the reason why sheep’s milk should be used in human nutrition. In seasonal animals, such as sheep, chemical composition of milk is significantly affected by environmental factors, especially climatic conditions and season (photoperiod length) [1,3]. In the studies carried out by Maria-Levrino and Gabina [11] it was shown that the season in which lambs were reared has a significant impact on protein and fat content in the ewes’ milk. Research has shown also that fat content in the milk of ewes that lambed in autumn was higher than in the milk of those that lambed in winter [12,13]. Our own studies conducted so far show that length of day affects both milk yield and chemical composition of sheep’s milk [5,14]. Since sheep’s milk is a product with high health-promoting value, it is important to investigate whether variation in length of the melatonin signal might modulate the milk’s chemical composition and its pro-health qualities. The aim of the study was to determine the effect of photoperiod length and treatment with exogenous melatonin on milk yield and chemical composition of sheep’s milk. An important hypothesis was to verify whether the introduction of exogenous melatonin during pregnancy (6 weeks before lambing) would contribute to the onset of melatonin-resistance in sheep and cause changes in chemical composition of milk [3].

## 2. Material and Methods

### 2.1. Animals and Diets

Sixty ewes of the Polish Longwool breed (a breed showing seasonal anestrus, body weight 65–70 kg, fertility about 180%) aged 3–4 years were used in the experiment. The animals were farmed at the Experimental Station of the Faculty of Animal Sciences of the University of Agriculture in Krakow (Poland) under natural lighting conditions (50°04′ N, 19°57′ E).

They were fed according to the physiological status based on the standards of the National Research Institute of Animal Production (Krakow-Balice, Poland) (Norms, 1993) [15]. From the 4th month of pregnancy, sheep were fed forage pasture 6 kg/per sheep/day (dry matter: 214 g, crude protein: 49 g, net energy: 1.24 MJ, per kg), grass silage-withered forage (4 kg/per sheep/day (dry matter: 382 g, crude protein: 58 g, net energy: 1.95 MJ, per kg) and hay ad libitum (dry matter: 882 g, crude protein: 185 g, net energy: 3.24 MJ, per kg). 

The unification of nutrition from the 5th month of pregnancy to the end of lactation was applied in order to eliminate the impact of nutrition on milk yield and lactation parameters [16]. Accordingly, sheep received 1.5 kg of pelleted granulate complete feed (crude protein: 220 g and net energy: 7.5 MJ, per kg containing only natural components- cereal grains, rape, dried legume plants, dried beet pulp, corn flour) produced under the name CJ (complete feed) by Polish company, and hay supplement (dry matter: 882 g, crude protein: 185 g, net energy: 3.24 MJ, per kg). The total daily ration was divided into two portions, morning (750 g CJ, hay ad libitum/per sheep) and evening (750 g CJ, hay ad libitum/per sheep). The hay was collected in the first cut before flowering from the same meadow (Experimental Station). All animals had free access to water and mineral licks. Sheep were weighed before mating, after weaning (57th day of lactation) and at the end of lactation. The zoohygienic and weather conditions were the same for all animals and body condition score was 3 or 4 (scale 0–5) [16].

### 2.2. Experimental Design

Estrus synchronization in the whole flock was performed using the Chronogest-C method. Specifically, polyurethane sponges impregnated with 40 mg cronolone (Intervet Holland, Warszawa, Poland) were inserted intravaginally for 14 days and then removed at the time of injection of 500 IU of pregnant mare’s serum gonadotropin (PMSG) (Folligon Intervet Holland, Warszawa, Poland). Estrus occurred within 24–48 h of PMSG administration, and the duration of estrus was additionally controlled using a teaser ram. Mating after estrus synchronization was performed at the beginning of September and in mid-January. Sixty (*n* = 60) sheep mothers rearing twins were selected and randomly divided into three treatment grousaps of 20 animal each: Group 1 that would lamb in February (G1—treatment group, *n* = 20), Group 2 lambing in June (G2–treatment group, *n* = 20, living in natural day-length conditions), and Group 3 lambing in June and treated with subcutaneous melatonin implants (G3—treatment group, *n* = 20). Lambs stayed with their mothers until 56 days of age, after which they were weaned, and ewes were used for milking. When the offspring were being reared, milk production of sheep was estimated based on weight gain of the lambs from 2 to 28 days of age, using a conversion factor of 4.5 l of milk per kg weight gain of lamb. The lambs were left with their mothers, they were fed only with milk [5]. Creep feeding was not used during the first 28 days of lamb rearing. The lambs were left with their mothers, they were fed only with milk. The addition of grain meal and hay was introduced after 28 days age. After weaning (from the 57th day of lactation), all ewes were milked twice a day (at 8:00 and 18:00 h), using an Alfa-Laval milking machine. The individual measurements of milk yield were carried out for morning and evening milking, every 10 days. The dry period was defined for every sheep, when milk yield was < 50 mL of milk per day. The lactation parameters such as: milk yield, the number of milking days and the total length of lactation (number of nursing and milking days) were monitored individually. Milk yield was recorded individually at 10-day intervals. G1 was milked in the period (April–August), G2 was milked in the period (August–October) and G3 was milked in the period (August–November). To determine the chemical composition of milk pooled milk samples every 28 days from each group of sheep. Group 1 has undergone five samplings, while G2 and G3–three sample collections each. Subcutaneous implants containing melatonin (18 mg per animal; Melovine, CEVA Sante Animal, Libourne, France–2,3) were implanted into ewes of the G3 group in the third month of pregnancy, six weeks before parturition (May). Treatment with melatonin implants was repeated after 90 days (August) to induce refractoriness to melatonin signal [2,3,4,5,14]

### 2.3. Analytical Methods 

Chemical composition was analysed according to the method developed by Budsławski [17]. Dry matter analysed for methods [AOAC 1995] and protein for methods by Kjeldahl [PN-68/A-86122]. The fat content was analysed used to the methods Gerber [PN-68/A-86122]. The lactose was analysed used to the methods Bertrand [PN-68/A-86122]. The calcium and phosphorus were determined using the AAS flame methods with spectrometer parameters AA240 FS [AOAC 1995] [2,3,4,5,14,17]. Fatty acid content was determined with gas chromatography (chromatograph PYE-UNICAM series 104 with chromatography column SUPELCOWAX 10.30 m., ø 0.53 mm., 1.0 µm) as described by Mann [18,19]. 

### 2.4. Statistical Analysis

The body weight and milk yield data were analyzed using the two-way repeated-measures analysis of variance (ANOVA, Statistica; StatSoft, Inc., Tulsa, OK, USA). The within-subjects factor was time and the between-subjects factor was treatment.

After the ANOVA, the Tukey post-hoc test was performed, when appropriate. The Kruskal–Wallis test followed by multiple comparisons of average ranks (Statistica; StatSoft, Inc., Tulsa, OK, USA) was used to determine the significance of the differences in milk yield and chemical compositions among the groups. Differences were considered significant at *p* < 0.05 with tendencies discussed at *p* ≤ 0.05 ≤ *p* ≤ 0.01. All data are expressed as means ± SEM. In the tables, differences, between groups are indicated using letters a,b for *p* ≤ 0.05 A,B for *p* ≤ 0.01.

## 3. Results 

### 3.1. Body Weight and Milk Yield

No weight differences were found within and between the groups. All sheep were weight-balanced (60.2 ± 0.3 kg) before mating. After weaning, the weight of sheep in each group was: Group 1—60.5 ± 0.4 kg, Group 2—59.3 ± 0.3 kg; Group 3—61.0 ± 0.4 kg, and at the end of lactation: Group 1—61.5 ± 0.4 kg, Group 2—60.5 ± 0.3 kg; Group 3—62.5 ± 0.5 kg. 

The study showed differences in milk yield during the rearing period of lambs. In the first 28 days of lactation, the highest milk yield was recorded for ewes of Group 1 (63.2 ± 13.9 L). The lowest milk yield (*p* ≤ 0.01) was found for Group 3 (44.6 ± 11.4 L). After lamb weaning (from day 57 until the end of lactation) the highest milk yield was found in animals of Group 1 (37.8 ± 8.1 L), while the lowest yield was recorded for Group 3 mothers (29.2 ± 7.6 L)—A significant (*p* ≤ 0.05) difference. The longest milking duration (117 ± 17.4 days) was noted in Group 1 ewes, with significantly (*p* ≤ 0.05) shorter period of milking for the animals of Group 3 (95 ± 10.4 days) and Group 2 (80 ± 9.8 days) (Table 1). The longest lactation was recorded for Group 1 (172 ± 18.3 days), with Group 2 ewes lactating the shortest (136 ± 10.4 days), a significant difference (*p* ≤ 0.05). Lactation of animals in Group 3 lasted (147 ± 12.1 days) and was 11 days longer than in Group 2 ewes (*p* ≤ 0.05) (Table 1). 

### 3.2. Chemical Composition

Significantly (*p* ≤ 0.05) least dry matter was assayed in the milk of Group 1 ewes. The highest content of dry matter was found in the milk of animals in Groups 3 and 2 (Table 2). In the first sampling, significantly (*p* ≤ 0.05) higher levels of protein were recorded in the milk of Group 2 (6.7 ± 0.8%) and Group 3 sheep (6.3 ± 0.8%). Protein content in the milk of Group 1 ewes was (5.42 ± 0.7%). In the second batch of samples, statistically significantly (*p* ≤ 0.05) higher protein content was found in the milk of ewes in Group 2 (7.49 ± 0.9%) and Group 3 (7.19 ± 0.9%). In this sampling, the milk of Group 1 sheep (5.7 ± 0.5%) was the poorest in proteins. In the third sample batch collected, the highest (*p* ≤ 0.01) protein content was recorded for the milk of Group 2 (8.05 ± 0.9%) and Group 3 animals (7.19% ± 0.9%). The protein content in Group 1 milk in this sampling was 5.49 ± 0.6%. (Table 3).

In the first sampling, significantly (*p* ≤ 0.05) higher fat content was identified in the milk of Group 3 (7.1 ± 1.2%) and Group 2 sheep (7.0 ± 1.3%). In this batch of samples, the milk of Group 1 ewes contained significantly less fat (6 ± 1.2%). In the second sampling, the most fat was recorded in the milk of Group 3 sheep (7.35 ± 0.9%) and the least amount (*p* ≤ 0.01) in the milk of animals in Group 1 (5.5 ± 0.8%). In the third batch of samples collected, the milk of Group 3 ewes was richest in fat (7.22 ± 0.8%), while the lowest (*p* ≤ 0.05) fat content was recorded in the samples collected from Group 1 (5.9 ± 0.9%) (Table 4). The milk obtained from Group 3 ewes had the highest (*p* ≤ 0.05) lactose content (Table 5). Different lactation time (season) and introduction of exogenous melatonin did not cause significant differences in the calcium content in sheep’s milk. Only in the third sampling, significantly (*p* ≤ 0.01) higher calcium content was noted for Group 3 sheep (239.9 ± 12.4 mg), while the milk of Group 2 sheep was the poorest in this mineral (193.5 ± 10.4 mg) (Table 5).

In the first batch of samples, the highest (*p* ≤ 0.01) phosphorus content was found in the milk of Group 3 mothers (167 ± 10.1 mg), the lowest - in the samples obtained from Group 2 and Group 1 (130 ± 9.8 mg and 131 ± 8.9 mg respectively). In the second sampling, significantly (*p* ≤ 0.01) higher levels of phosphorus were found in the milk of Group 3 animals (159 ± 10.3 mg), while the milk of Group 1 showed the lowest phosphorus content (139 ± 9.2 mg). In the final, third sampling, the highest (*p* ≤ 0.01) phosphorus concentration was found in the milk collected from Group 3 (179 ± 10.2 mg). The samples from ewes in Group 1 contained less phosphorus (151 ± 10.1 mg), with the lowest value recorded in the milk from Group 2 animals (147 ± 8.7 mg) (Table 5).

### 3.3. Fatty Acids

The highest (*p* ≤ 0.01) content of C4:0 and C6:0 acids was found in the milk of Group 1 ewes, and the lowest in the milk of Group 3 animals. The highest (*p* ≤ 0.01) levels of C10:0 and C12:0 acids were recorded for the milk obtained from Group 2, the lowest - in the milk collected from ewes in Group 1. The milk of Group 3 animals was the richest (*p* ≤ 0.01) in C14:0 acid (2.16 ± 2.01%), while the content of this acid was the lowest in the milk of treatment Group 1 (12.68 ± 1.36). The highest (*p* ≤ 0.05) concentration of acid C16:0 was noted in the milk of Group 3 ewes (28.39 ± 1.49%) and the lowest—in the samples collected from Group 1 (25.62 ± 2.08). The highest content of C18:0 acid was recorded in the milk from animals in Group 1 (8.54 ± 1.34%), the lowest - in the samples from Group 2 (5.52 ± 0.87%) and Group 3 (5.80 ± 1.63) (Table 6). The highest (*p* ≤ 0.01) levels of C10:1, C14:1, and C16:1 fatty acids were found in the samples obtained from Groups 2 and 3, the milk from Group 1 ewes being the poorest in these fatty acids. The highest (*p* ≤ 0.01) content of C18:1 (*n* = 9), C18:1 (*n* = 7) and C18:1 (cis-12) was demonstrated in the milk obtained from animals in Group 1, while the lowest was noted in the milk from sheep in Group 3. Significantly (*p* ≤ 0.05) higher concentrations of C20:1 acid were noted in the milk of Group 1 (0.09 ± 0.02%) and Group 3 ewes (0.05 ± 0.01%) (Table 7). The samples collected from treatment Group 1 contained the most (*p* ≤ 0.05) C18:2 acid (2.58 ± 0.45%), the milk of Group 3 ewes being the poorest in this component (1.89 ± 0.25%). The highest (*p* ≤ 0.01) C18:3 acid content was found in the milk of Group 1 sheep (0.63 ± 0.13%), with lower content recorded in the samples from Group 2 (0.46 ± 0.03) and Group 3 (0.38 ± 0.01%). Analysis of milk samples obtained from the treatment groups showed that the largest concentration of conjugated linoleic acid (CLA) was present in the milk of sheep from Group 1 (0.87 ± 0.16%). Significantly (*p* ≤ 0.01) lower CLA content was noted in the milk of sheep in Group 2 (0.49 ± 0.10) and Group 3 (0.41 ± 0.09) (Table 8).

## 4. Discussion

In analysis of milk yield from the milking period, it was shown that the most milk was produced by Group 1 sheep and the least by Group 2 animals. Similar results were obtained by Molik et al. [2,5]. This previous research demonstrated that ewes milked in the period of shortening day produced 50% less milk than animals that lambed in the spring and summer. Research by Molik at al. [3] showed that the introduction of a variable melatonin signal in sheep modulates content of SFA (saturated fatty acid) decreased and the content of PUFA (polyunsaturated fatty acid) increased. Implantation of exogenous melatonin and exposition of ewes to artificial short days (16D:8L) caused a decrease in MUFA (monounsaturated fatty acid) and PUFA content. The study showed that the introduction of biochemical and light modulation of photoperiod through increased melatonin secretion via subcutaneous implants and an artificial short-day condition can affect milk composition in seasonally breeding ewes. Melatonin not only decrease lactose concentration but also caused a significant deterioration in the fatty acid profile of milk. These results imply seasonal variations in milk composition common to Polish milking ewes, which cannot be overridden by farming though diet or breeding system. Further studies are needed to confirm that phenomenon in order to make sheep milk production system more regular around the year together with better manufacturing properties of milk [3]. Also, the research results being presented here showed that introduction of exogenous melatonin implants in Group 3 sheep 6 weeks before lambing led to reactiveness to melatonin (melatonin resistance), and lactation in this Group lasted 11 days longer than in ewes of Group 2. Throughout the study, it was found that the dry matter content in the milk of Group 1 was lower than in the milk obtained from ewes that lambed in June (Group 2) and those treated with exogenous melatonin (Group 3). Sheep milked in short photoperiod conditions (Group 2 and Group 3) from the beginning of lactation produced milk with a higher dry matter content than sheep of Group 1. In all examined groups of sheep, the content of dry matter was increasing over the course of the lactation period, which had also been confirmed by previous studies [5]. Protein content analysis determined that the milk of mothers in Group 2 and Group 3 presented a higher protein content than the milk from Group 1 animals. In addition, the milk of sheep from Groups 2 and 3 already in the first month of milking contained more protein than the milk of ewes in Group 1 [20,21,22].

Analysis of changes in fat content in sheep’s milk showed that the milk obtained from Group 2 and Group 3 animals was characterized by a higher fat content compared to the milk collected from animals in Group 1. According to the study by Pavić et al. [22] fat content increases over the course of lactation. The results collected showed that lactose content in the milk of Group 2 sheep was significantly lower compared to the content of this component in the milk from animals of Group 1. Over the course of lactation, lactose levels in milk systematically decreased in all sheep groups. Similar results regarding changes in lactose concentration in sheep’s milk were obtained by Bonczar and Reguła [23] as well as Park et al. [24]. Chemical composition of the milk from ewes in Groups 2 and 3 in the first months of milking was similar to that of the milk from Group 1 sheep over the last two months of milking. Different lambing time (season) and administration of external melatonin did not cause significant changes in calcium content. Only in the third batch of samples, the milk from ewes with melatonin implants (Group 3) was characterized by a higher calcium content in relation to sheep that lambed in February. However, the overall level of calcium in the milk of all the treatment groups was higher than that found in the studies by other authors [9]. Introduction of melatonin implants had a significant effect on phosphorus content. The milk of sheep milked during short day (Groups 2 and 3) was characterized by a significantly higher content of this component. Content of phosphorus in the milk of sheep that lambed in February (Group 1) was comparable to that determined by Haenlein [9]. In the fatty acids content assays, it was determined that the milk of Group 1 ewes had a significantly higher content of C4:0, C6:0, C17:0 and C18:0 acids. In contrast, the milk from sheep in Groups 2 and 3 was richer in C8:0, C 10:0, C12:0, C14:0, and C16:0 fatty acids compared to that of Group 1 sheep. In general, the highest content of SFAs was recorded in the milk of Group 3 animals. The SFA levels recorded in the milk of Group 1 sheep were similar to results obtained in the studies by Carta et al. [25]. Based on the results from MUFA acid assays, the highest concentration of these acids was present in the milk of Group 1 ewes. The milk of animals in Groups 2 and 3 contained significantly less C18:1 (*n* = 9), C18:1 (*n* = 7) and C18:1 (cis-12) acids. The obtained results align with the research carried out by Meluchova et al. [7], Carta et al. [25], and Molik et al. [3]. The highest content of PUFAs in the experiment was recorded in the milk of sheep in Group 1. The lowest levels of CLA were determined for the milk of treatment Groups 2 and 3. Taking into account the health-promoting and nutritional properties of polyunsaturated fatty acids (PUFAs), it can be assumed based on the research results presented that the milk obtained from ewes in Groups 2 and 3 offers weaker pro-health parameters. The values for linoleic acid recoded in the study are comparable to the results obtained in the studies of Luna et al. [26], Clare et al. [27] Meluchova et al. [7]. Sheep milk offers exceptional health benefits; it owes its properties to the rich chemical composition. This high health value of sheep’s milk results from the presence of inter alia antioxidant substances such as CLA. Conjugated linoleic acid (CLA) is one of the most important antioxidants of milk fat and exhibits anti-carcinogenic and anti-mutagenic effects [28]. Recently, we observe an increase in popularity of the so-called functional foods, i.e., foodstuffs demonstrating health-promoting properties, resulting mainly from the presence of bioactive substances that have a positive effect on metabolic processes and correct balance of various components [28]. Currently, also the interest in the possibility of using sheep’s milk in treatment of metabolic disorders in humans is growing [29,30]

## 5. Conclusions

The research results presented here showed that Group 1 ewes lambed in February were characterized by longer lactation and higher milk yield in comparison to ewes in Group 2, that lambed in June and Group 3, that lambed in June and was treated with exogenous melatonin. The milk of animals in Groups 2 and 3 showed significantly higher content of dry matter, protein, and fat, as well as higher levels of saturated fatty acids (SFA) compared to the milk of Group 1 sheep. Group 1 sheep produced milk with significantly more MUFA and PUFA fatty acids than mothers in treatment Groups 2 and 3. In particular, the milk of Group 1 animals was richest in CLA, whereas the milk of Group 3 animals presented the lowest content of this component. The research has thus shown that melatonin signal strongly modulates sheep’s milk yield and chemical composition of milk. The obtained results show that optimal health benefits are offered by milk obtained in short photoperiod conditions, i.e., during short-day melatonin signaling.

## Figures and Tables

**Table 1:** The lactation parameters in sheep from the Group 1 (lambing in February–milked from April to August, Group 2 (lambing in June–milked from August to October), Group 3 (lambing in June–milked from August to November and melatonin-treated before 6 weeks parturition).

Parameters of Lactation	G1	G2	G3	SEM
Milk yield of the lamb-nursing period (liter)	63.2 ^B^	61.08 ^B^	44.62 ^A^	10.8
Days of milking (days)	117 ^A^	80 ^B^	95 ^B^	11.2
Length of lactation (days)	172 ^A,B^	136 ^A,a^	147 ^B,b^	12.4
Milk production during lactation (liter)	37.8 ^A,B,a^	30.2 ^A,b^	29.2 ^B^	9.3

Parameters of lactation: milk yield of the lamb-nursing period the number of milking days and the total length of lactation, total milking production—column header; G1—Group lambing in February (milked from April to August)–column header; G2—Group lambing in June (milked from August to October)–column header; G3—Group-lambing in June (milked from August to November and melatonin-treated before 6 weeks parturition)–column header; ^a, b^—means within a row with different lowercase superscripts differ significantly at (*p* < 0.05); ^A, B^—means within a row with different lowercase superscripts differ significantly at (*p* < 0.01).

**Table 2:** Changes in dry matter content (%) in sheep’s milk from the Group 1 (lambing in February–milked from April to August, Group 2 (lambing in June–milked from August to October), Group 3 (lambing in June–milked from August to November and melatonin-treated before 6 weeks parturition).

Number of Sampling	G1	G2	G3	SEM
1	16.96 ^A,a^	18.16 ^B^	18.52 ^b^	4.2
2	16.42 ^a^	18.68 ^b^	19.65 ^b^	4.3
3	16.84 ^a^	19.7 ^b^	19.71 ^b^	4.6
4	16.4	-	-	6.5
5	19.84	-	-	6.7

Number of collection in sheep’s milk during the lactation–column header; G1—Group lambing in February (milked from April to August)–column header; G2—Group lambing in June (milked from August to October)–column header; G3—Group-lambing in June (milked from August to November and melatonin-treated before 6 weeks parturition)–column header; ^a, b^—means within a row with different lowercase superscripts differ significantly at (*p* < 0.05); ^A, B^—means within a row with different lowercase superscripts differ significantly at (*p* < 0.01).

**Table 3:** Changes in protein (%) content in sheep’s milk from the Group 1 (lambing in February–milked from April to August, Group 2 (lambing in June–milked from August to October), Group 3 (lambing in June–milked from August to November and melatonin-treated before 6 weeks parturition).

Number of Sampling	G1	G2	G3	SEM
1	5.42 ^a^	6.7 ^b^	6.3 ^b^	0.8
2	5.7 ^a^	7.49 ^b^	7.19 ^b^	0.7
3	5.49 ^A^	8.05 ^B^	7.6 ^B^	3.2
4	6.2	-	-	0.8
5	6.86	-	-	0.8

Number of collection in sheep’s milk during the lactation–column header; G1—Group lambing in February (milked from April to August)–column header; G2—Group lambing in June (milked from August to October)–column header; G3—Group-lambing in June (milked from August to November and melatonin-treated before 6 weeks parturition)–column header; ^a, b^—means within a row with different lowercase superscripts differ significantly at (*p* < 0.05); ^A, B^—means within a row with different lowercase superscripts differ significantly at (*p* < 0.01).

**Table 4:** Changes in fat (%) content in sheep’s milk from the Group 1 (lambing in February–milked from April to August, Group 2 (lambing in June–milked from August to October), Group 3 (lambing in June–milked from August to November and melatonin-treated before 6 weeks parturition).

Number of Sampling	G1	G2	G3	SEM
1	6.0 ^a^	7.0 ^b^	7.1 ^b^	1.2
2	5.5 ^A^	6.65 ^B^	7.35 ^B^	0.9
3	5.9 ^a^	6.51 ^b^	7.22 ^b^	0.8
4	5.6	-	-	0.9
5	8.4	-	-	2.1

Number of collection in sheep’s milk during the lactation–column header; G1—Group lambing in February (milked from April to August)–column header; G2—Group lambing in June (milked from August to October)–column header; G3—Group-lambing in June (milked from August to November and melatonin-treated before 6 weeks parturition)–column header; ^a, b^—means within a row with different lowercase superscripts differ significantly at (*p* < 0.05); ^A, B^—means within a row with different lowercase superscripts differ significantly at (*p* < 0.01).

**Table 5:** Changes in lactose (%), calcium (%), and phosphorus (%) content in sheep’s milk from the Group 1 (lambing in February–milked from April to August, Group 2 (lambing in June–milked from August to October), Group 3 (lambing in June–milked from August to November and melatonin-treated before 6 weeks parturition).

Number of Sampling	G1	G2	G3	SEM
Lactose (%)				
1	4.7 ^a^	4.07 ^a^	5.18 ^b^	0.5
2	4.57 ^a^	3.72 ^b^	4.28 ^b^	0.6
3	4.54 ^A^	2.99 ^B^	4.1 ^A^	0.7
4	4.6	-	-	0.8
5	4.32	-	-	1.9
Calcium (%)				
1	209.8	206.4	196.6	9.2
2	204.7	212.9	218.6	9.3
3	198.9 ^B^	193.5 ^B^	239.9 ^A^	9.4
4	206.1	-	-	8.9
5	197.2	-	-	8.6
Phosphorus (%)				
1	131.0 ^B^	130 ^B^	167.0 ^A^	9.8
2	139.5	150.0	159	9.2
3	151.0 ^B^	147.0 ^B^	179.0 ^A^	8.7
4	144.0	-	-	9.8
5	134.0	-	-	9.1

Number of collection in sheep’s milk during the lactation–column header; G1—Group lambing in February (milked from April to August)–column header; G2—Group lambing in June (milked from August to October)–column header; G3—Group-lambing in June (milked from August to November and melatonin-treated before 6 weeks parturition)–column header; ^a, b^—means within a row with different lowercase superscripts differ significantly at (*p* < 0.05); ^A, B^—means within a row with different lowercase superscripts differ significantly at (*p* < 0.01).

**Table 6:** Total content of saturated fatty acids in sheep’s milk from the Group 1 (lambing in February–milked from April to August), Group 2 (lambing in June–milked from August to October), Group 3 (lambing in June- milked from August to November and melatonin-treated before 6 weeks parturition).

Saturated Fatty Acids (SFA) (%)	G1	G2	G3	SEM
C 4: 0	2.39 ^A,a^	2.07 ^b^	1.95± ^B,c^	0.13
C 6: 0	2.38 ^a^	2.22	2.01 ^b^	0.26
C 8: 0	2.68	2.77	2.42	0.27
C 10: 0	9.16 ^A^	11.23 ^B^	10.22	0.51
C 12: 0	5.37 ^A^	7.88 ^B^	7.65 ^B^	0.92
C 14: 0	12.68 ^A,a^	15.19 ^B^	15.84 ^b^	2.01
C 15: 0	1.07	1.1	1.13	0.05
C 16: 0	25.62 ^a^	26.38	28.39 ^b^	0.96
C 17: 0	0.48	0.44	0.43	0.07
C 18: 0	8.54 ^A^	5.52 ^B^	5.80 ^B^	2.5
C 20: 0	0.21	0.17	0.17	0.03
SFA	6.41 ^a^	6.84 ^b^	6.91 ^b^	1.38

Number of fatty acids in sheep’s milk during the lactation–column header; G1—Group lambing in February (milked from April to August)–column header; G2—Group lambing in June (milked from August to October)–column header; G3—Group-lambing in June (milked from August to November and melatonin-treated before 6 weeks parturition)–column header; ^a, b, c^—means within a row with different lowercase superscripts differ significantly at (*p* < 0.05); ^A, B^—means within a row with different lowercase superscripts differ significantly at (*p* < 0.01).

**Table 7:** Total content of unsaturated fatty acids in sheep’s milk from the Group 1 (lambing in February–milked from April to August); Group 2 (lambing in June–milked from August to October), Group 3 (lambing in June–milked from August to November and melatonin-treated before 6 weeks column header).

Monounsaturated Fatty Acids (MUFA) (%)	G1	G2	G3	SEM
C 10: 1	0.26 ^A^	0.46 ^B^	1.95 ^B^	0.3
C 14: 1	0.30 ^a^	0.43	2.01 ^b^	0.26
C 16: 1	1.12 ^a^	1.26	1.48 ^b^	0.39
C 17: 1	0.23 ^a^	0.23 ^a^	0.25 ^b^	0.01
C 18: 1(*n* = 9)	17.58 ^A^	15.68 ^B^	14.59 ^B^	2.02
C 18: 1 (*n* = 7)	2.62 ^A^	1.11 ^B^	1.32 ^B^	0.20
C 18: 1(cis = 12)	0.98 ^A,a^	0.46 ^B^	0.58 ^b^	0.16
C 20: 1	0.09 ^a^	0.06	0.05 ^b^	0.01
Total MUFA	2.9 ^A,a^	2.46 ^b^	2.39 ^B^	0.03

Number of fatty acids in sheep’s milk during the lactation–column header; G1—Group lambing in February (milked from April to August)–column header; G2—Group lambing in June (milked from August to October)–column header; G3—Group-lambing in June (milked from August to November and melatonin-treated before 6 weeks parturition)–column header; ^a, b^—means within a row with different lowercase superscripts differ significantly at (*p* < 0.05); ^A, B^—means within a row with different lowercase superscripts differ significantly at (*p* < 0.01).

**Table 8:** Global content of polyunsaturated fatty acids in sheep’s milk from the Group 1 (lambing in February–milked from April to August); Group 2 (lambing in June–milked from August to October), Group 3 (lambing in June–milked from August to November and melatonin-treated before 6 weeks parturition).

Polyunsaturated Fatty Acids (PUFA) (%)	G1	G2	G3	SEM
C 18: 2	2.58 ^a^	2.13	1.89 ^b^	0.28
C 18: 3	0.63 ^A,a^	0.46 ^c^	0.38 ^B,b^	0.01
CLA	0.87 ^A^	0.46 ^B^	0.41 ^B^	0.09
Total MUFA	1.36 ^A,a^	1.02 ^b^	0.89 ^B^	0.24

Number of fatty acids in sheep’s milk during the lactation—column header; G1—Group lambing in February (milked from April to August)–column header; G2—Group lambing in June (milked from August to October)–column header; G3—Group-lambing in June (milked from August to November and melatonin-treated before 6 weeks parturition)–column header; ^a, b, c^—means within a row with different lowercase superscripts differ significantly at (*p* < 0.05); ^A, B^—means within a row with different lowercase superscripts differ significantly at (p < 0.01). CLA: (Conjugated linoleic acid), MUFA: (monounsaturated fatty acid).
